# Association between systemic immune-inflammation index and psoriasis: a population-based study

**DOI:** 10.3389/fimmu.2024.1305701

**Published:** 2024-03-05

**Authors:** Xiya Zhao, Junqin Li, Xinhua Li

**Affiliations:** ^1^ Ninth Clinical College of Medicine, Shanxi Medical University, Taiyuan, China; ^2^ Department of Dermatology, Taiyuan Central Hospital, Shanxi Medical University, Taiyuan, China; ^3^ Key Laboratory of Stem Cells for Immunologic Skin Diseases, Taiyuan Central Hospital, Taiyuan, China

**Keywords:** systemic immune-inflammation index, psoriasis, PASI, NHANES, stroke

## Abstract

**Background:**

The systemic immune-inflammation index (SII),as measured by lymphocyte, neutrophil and platelet counts in peripheral blood, is regarded as a favorable indicator of both inflammatory state and immune response. Psoriasis is an immune-mediated disease notable for its chronic inflammation of the entire system. Our research sought to explore the latent link between psoriasis and SII.

**Methods:**

We performed a cross-sectional investigation utilizing data extracted from the National Health and Nutrition Examination Survey (NHANES, 2009-2014). Employing multivariate linear regression models and subgroup analysis, we sought to uncover the association between SII and psoriasis.

**Results:**

This study enrolled a total of 17,913 participants as part of its research cohort. Our multivariate linear regression analysis revealed a notable and positive correlation between SII and psoriasis [1.013 (1.000, 1.026)]. As SII tertiles increased, the risk of psoriasis demonstrated an upward trend. The significant dependence on this positive association were maintained in women, BMI(≥ 30 kg/m2),non-stroke and non-cancer subjects in subgroup analysis and interaction tests. Furthermore, we identified a significant association between SII and psoriasis, characterized by two consecutive inverted U-shaped patterns. Notably, the analysis revealed the most prominent inflection point at a specific value of 797.067.

**Conclusions:**

The results indicate a significant correlation between elevated SII levels and the presence of psoriasis. However, to corroborate and strengthen these results, additional large-scale prospective studies are required.

## 1 Background

Psoriasis, a genetic skin disorder mediated by the immune system, impacts approximately 2-3% of the global population (1, 2). It typically affects the skin, but may also affects different organ systems such as the joints (3). The risk factors of psoriasis can be divided into extrinsic(trauma, drugs, infections, vaccination, lifestyle)and intrinsic(obesity, diabetes mellitus, dyslipidemia, hypertension, mental stress)groups with a view to prevent (4). Innate and adaptive immune responses are involved in the progression of psoriatic inflammation (5). Antimicrobial peptides (AMPs), such as LL-37, β-defensin and S100 protein, trigger and maintain inflammatory pathways in psoriasis (6). In the maintenance phase of psoriasis, the TNF-α/IL-23/IL-17 axis assumes a crucial role (7).

Recent studies have reported that psoriasis shares an underlying chronic inflammatory basis with comorbidities such as metabolic syndrome and cardiovascular disease (8–11). Uncovering the role of adipose inflammation in psoriasis is being intensively investigated (12, 13). Biologics such as efolizumab can delay the onset of systemic inflammatory psoriasis (14, 15). Other studies have suggested that clinical parameters of plasma cytokines and inflammation may serve as novel strategies to monitor the progression of psoriasis (16, 17). Elevated levels of various inflammatory and immune-based indices, such as the neutrophil-to-lymphocyte ratio (NLR), platelet-to-lymphocyte ratio (PLR), and monocyte-to-lymphocyte ratio (MLR) have been observed and found to correlate with Psoriasis Area and Severity Index (PASI) scores in individuals with psoriasis (18, 19).

SII has become a prognostic biomarker for a variety of malignant tumors, including gastric cancer (20, 21), non-small cell lung cancer (22, 23), esophageal cancer (24, 25) and colorectal cancer (26). It provides a comprehensive reflection of the body’s equilibrium between inflammatory factors and immune responses. Besides, the new score has been established as an effective predictive indicator of cardiovascular events (27). Some recent findings suggest that elevated SII levels are associated with hepatic steatosis and increased urinary albumin excretion (28, 29). The differences in this study are, first, that the study population was a sample of NHANES and that the sample size was increased, as were confounders for psoriasis or general health. Second, SII and the likelihood of psoriasis were plotted as a curve-fitted graph for analysis.

As a result, our objective was to investigate the correlation between the SII and psoriasis within the United States population. To accomplish this, we utilized the NHANES dataset in this study.

## 2 Methods

### 2.1 Study population

**Figure 1:**
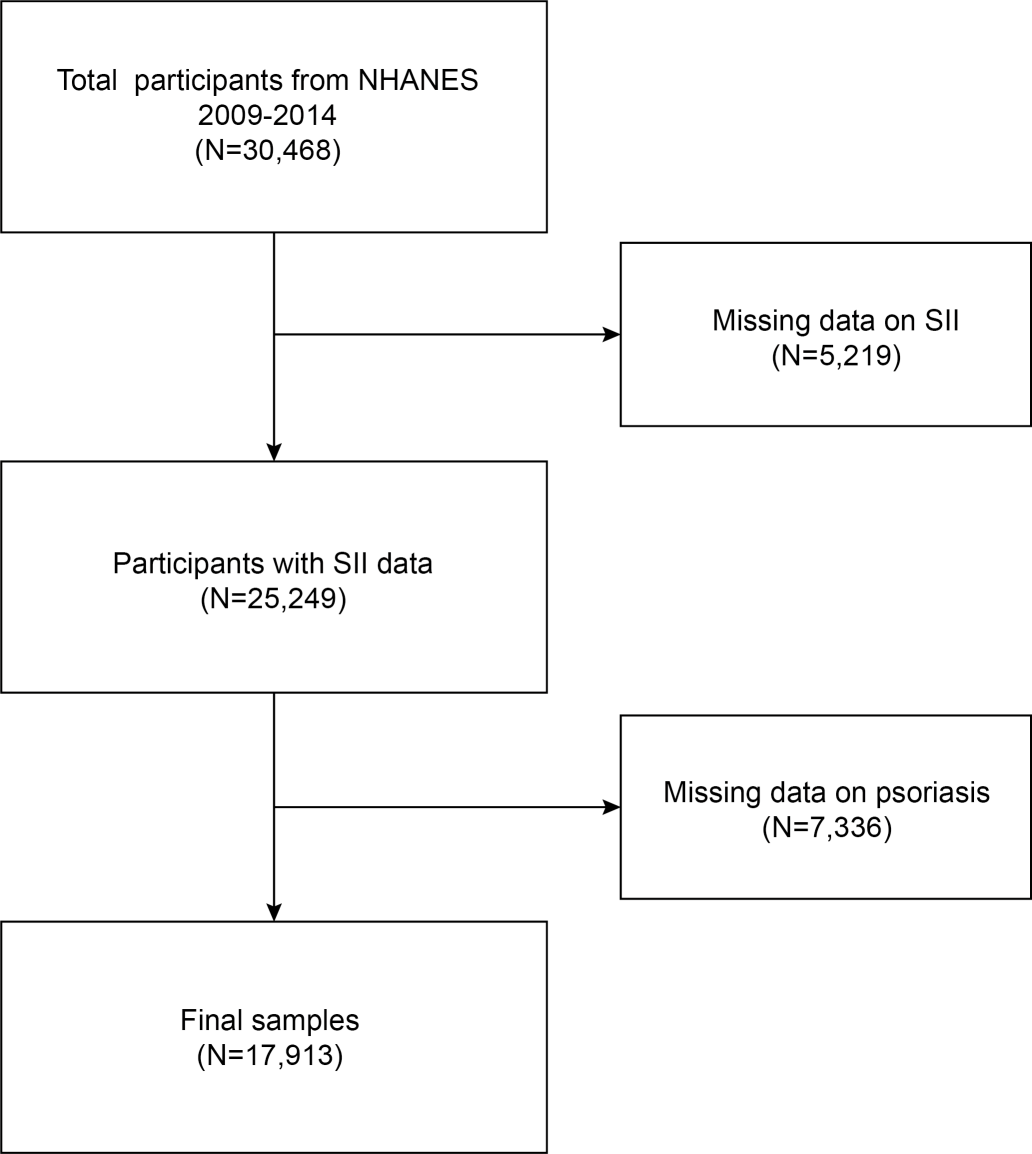
Flow chart of participants selection. NHANES, National Health and Nutrition Examination Survey.

The NHANES is a comprehensive survey conducted biennially by the National Center for Health Statistics (NCHS), providing cross-sectional data that represents the entire US population. The survey using the continuous cycle NHANES data set of 2009-2014.Initially, a cohort of 30,468 participants was enrolled for the study. We excluded 5,219 participants with missing SII data (neutrophil, lymphocyte, and platelet count) and 7,336 with missing psoriasis data. The study ultimately included 17913 eligible participants. **Figure 1** presents the visual representation of our sample selection process.

### 2.2 Definition of systemic immune-inflammation index

The Systemic Immunoinflammatory Index (SII), a new biomarker of local immune response and systemic inflammation throughout the body, has been shown to correlate with the prognosis of cancer patients. Regarded as a continuous variable, The calculation of SII for each participant was performed utilizing the formula:


$$SII=\frac{platelet\;count\times neutrophil\;count}{lymphocyte\;count}$$

Lymphocyte, neutrophil and platelet count using automatic hematology analysis equipment (Coulter ^®^ DxH analyzer 800) determination of complete blood count, expressed as x 103 cells/ml. we designated SII as the exposure variable and psoriasis condition as an outcome variable in this study. Since the effect size was not significant, SII/100 was used to amplify the effect size by a factor of 100.

### 2.3 Selection of covariates

The covariates considered in this study encompassed various factors, including sex (men/women), age (years), race (Mexican American/other Hispanic/non-Hispanic White/non-Hispanic Black/other races), educational attainment (less than high school, high school or equivalent/above high school), body mass index (BMI, kg/m2), smoking status (yes/no), alcohol consumption (yes/no), presence of coronary artery disease (yes/no), diabetes mellitus (yes/no), congestive heart failure (yes/no), history of stroke (yes/no), and presence of cancer (yes/no). Individuals were categorized into three groups based on their body mass index (BMI):<25 kg/m2 indicating normal weight, 25-29.9 kg/m2 indicating overweight, and ≥30 kg/m2 indicating obesity.

### 2.4 Statistical analysis

The statistical analyses were carried out following the guidelines of the Disease Control and Prevention (CDC), taking into account the appropriate NHANES sampling weights and employing complex multi-stage cohort surveys. Continuous variables are expressed as means with standard deviation (SD) and categorical variables as proportions. We used multivariate logistic regression analysis between SII and psoriasis to construct multivariate tests that resulted in beta values and 95% confidence intervals. The multivariate test involved three models: model 1 (unadjusted), model 2 (adjusted for gender, age, and race), and model 3 (adjusted for all covariates).Smooth curve fitting was performed simultaneously by adjusting the variables. A threshold effect analysis model was used to investigate the relationship and inflection point between SII and psoriasis. Subgroup analysis of the SII-psoriasis relationship was conducted considering stratification factors including gender (man/woman), BMI (normal weight/overweight/obesity), stroke (yes/no), cancer (yes/no). In the process, we used the statistical calculation and graphics software R(version 4.1.3) and the Irrigation Statistics Software (version 2.0) for statistical research. Significance was established at a threshold of P< 0.05.

## 3 Results

### 3.1 Baseline characteristics

This study involved 17913 participants with a mean age of (46.02 ± 19.26) years. Of these, 48.67% were men and 51.33% were women. Among these participants, 14.98% were Mexican American, 9.84% were other Hispanic,41.87% were non-Hispanic white, 22.33%were non-Hispanic black, and 12.10% were from other races. The mean SII score was (520.20 ± 388.94) for all participants. In comparison to the no history of psoriasis group, the history of psoriasis group is significantly more likely to be older and have comorbidities including stroke, coronary artery disease,diabetes,congestive heart failure and cancer with a higher proportion of non-Hispanic white and education above high school; with higher smoking and alcohol status; and higher levels of BMI and SII.The statistical analysis did not demonstrate a significant difference in gender and stroke between the two groups (p > 0.05) (**Table 1**).

**Table 1:** Baseline characteristics of patients with and without psoriasis.

Variable	History of Psoriasis (n = 474 [2.6%])	No History of Psoriasis (n = 17439 [97.4%])	*P* Value
Age (years)	50.970 ± 17.662	45.885 ± 19.288	<0.001
Gender (%)			0.802
Men	48.101	48.684	
Women	51.899	51.316	
Race (%)			<0.001
Mexican American	8.650	15.150	
Other Hispanic	9.283	9.851	
Non-Hispanic White	59.072	41.407	
Non-Hispanic Black	10.549	21.504	
Other Races	12.447	12.088	
Education level(%)			0.003
Less than high school	24.262	32.192	
High school or GED	22.363	19.869	
Above high school	53.376	47.830	
Others	0.000	0.109	
Smoking(%)	56.863	43.464	<0.001
Alcohol use(%)	22.432	16.746	0.038
BMI (kg/m2)	29.829 ± 7.058	28.676 ± 7.017	<0.001
Comorbidities(%)			
Diabetes	16.667	11.038	<0.001
Congestive heart failure.	5.470	2.996	<0.001
Coronary artery disease	7.659	3.800	<0.001
Stroke	4.376	3.634	0.603
Cancer	14.880	9.027	<0.001
SII (1,000 cells/µl)	577.261 ± 320.458	518.651 ± 390.534	<0.001

Mean ± SD for continuous variables: the p-value was calculated by weighted linear regression model. % for categorical variables: the p-value was calculated by a weighted chi-square test. BMI, body mass index; SII, systemic immune-inflammation index.

### 3.2 Association between systemic immune-inflammation index and psoriasis

Our findings suggest that the higher the SII, the greater the likelihood of developing psoriasis. This association remained significant in both our crude model (OR=1.016; 95% CI, 1.003–1.029, p<0.05) and minimally adjusted model (OR=1.013; 95% CI, 1.000–1.026, p<0.05). However, after adjusting all covariates, this significant positive correlation became insignificant in model 3 (OR=1.009; 95% CI, 0.992–1.026,p>0.05). We further transformed SII for sensitivity analysis from continuous variables to categorical variables (quartiles). Participants in the highest quartile (OR=1.645; 95% CI, 1.261–2.145, p<0.05) of SII had a statistically significant 64.5% increased risk of developing psoriasis when contrasted with those situated in the lowest SII quartile. Participants belonging to SII quartile 2 and 3 displayed an increased risk of psoriasis in comparison to the lowest quartile; however, this association did not attain statistical significance (**Table 2**).

**Table 2:** The association between SII and psoriasis.

	Model 1	Model 2	Model 3
	OR(95% CI) P value	OR(95% CI) P value	OR(95% CI) P value
SII/100	1.016 (1.003, 1.029) 0.01702	1.013 (1.000, 1.026) 0.04364	1.009 (0.992, 1.026) 0.32130
SII quartiles			
Quartile 1	Reference	Reference	Reference
Quartile 2	1.174 (0.881, 1.565) 0.27441	1.084 (0.812, 1.448) 0.58442	1.035 (0.740, 1.448) 0.84087
Quartile 3	1.315 (0.993, 1.741) 0.05616	1.159 (0.873, 1.539) 0.30870	1.078 (0.777, 1.494) 0.65331
Quartile 4	1.944 (1.497, 2.524)<0.00001	1.645 (1.261, 2.145) 0.00024	1.433 (1.052, 1.952) 0.02263
P for trend	<0.00001	0.00003	0.00750

Model 1, no covariates were adjusted. Model 2, age, sex, and race were adjusted. Model 3, age, sex, race, education level, smoking status, drinking status, BMI, diabetes, congestive heart failure,coronary artery disease, stroke and cancer were adjusted.

Multivariate logistic regression analysis was used to calculate beta values and 95% confidence intervals.

95% CI, 95% confidence interval; OR, odds ratio; SII, systemic immunity-inflammation index.

p< 0.05 was considered statistically significant.

Further subgroup analysis revealed inconsistent associations between SII and psoriasis, as shown in **Table 3**. In subgroup analyses by gender stratification, our results revealed an independent and significantly positive association between SII and psoriasis exclusively among women (OR = 1.031; 95% CI, 1.002−1.061, p<0.05) but not statistically significant in all models for men. When examining subgroups by degree of body mass index, we observed a robust positive correlation between SII and the obese group (≥ 30 kg/m2) in both the unadjusted and partially adjusted models. A significant relationship between SII with psoriasis was detected in non-cancer subjects (OR = 1.014, 95% CI 1.001−1.026, p<0.05). The interaction test indicated that no significant dependence was observed for the positive correlations between sex, age, body mass index, and cancer with SII and psoriasis (p for interaction >0.05).Only in the stroke stratification the association between SII and psoriasis was significantly different.

**Table 3:** Subgroup analysis for the association between SII and psoriasis.

SII/100	Model 1	Model 2	Model 3
	OR (95% CI) P value	OR (95% CI) P value	OR (95% CI) P value
gender			
Men	1.011 (0.998, 1.025) 0.1012	1.009 (0.993, 1.026) 0.2843	1.005 (0.979, 1.031) 0.7196
Women	1.038 (1.010, 1.066) 0.0066	1.031 (1.002, 1.061) 0.0339	1.024 (0.985, 1.065) 0.2262
P for interaction	0.1100	0.2021	0.4107
Race			
Mexican American	0.967 (0.860, 1.088) 0.5814	0.970 (0.863, 1.090) 0.6062	0.968 (0.849, 1.102) 0.6222
Other Hispanic	1.070 (0.980, 1.167) 0.1298	1.070 (0.980, 1.168) 0.1310	1.064 (0.951, 1.192) 0.2800
Non-Hispanic White	1.036 (1.008, 1.064) 0.0110	1.030 (1.002, 1.059) 0.0352	1.026 (0.994, 1.058) 0.1091
Non-Hispanic Black	1.029 (0.976, 1.085) 0.2832	1.024 (0.972, 1.080) 0.3716	1.1 (0.900, 1.114) 1.2 0.9866
Other Races	1.031 (0.966, 1.102) 0.3551	1.031 (0.966, 1.100) 0.3639	1.028 (0.945, 1.117) 0.5202
P for interaction	0.2210	0.3125	0.6347
BMI			
<25	1.023 (0.981, 1.066) 0.2857	1.008 (0.959, 1.059) 0.7635	1.008 (0.955, 1.063) 0.7736
>=25,<30	1.010 (0.995, 1.024) 0.1851	1.011 (0.994, 1.028) 0.2126	1.007 (0.982, 1.032) 0.5833
>=30	1.056 (1.019, 1.094) 0.0024	1.045 (1.007, 1.085) 0.0201	1.027 (0.982, 1.074) 0.2358
P for interaction	0.0857	0.2823	0.7365
STROKE			
YES	0.824 (0.678, 1.002) 0.0518	0.803 (0.659, 0.978) 0.0290	0.830 (0.669, 1.031) 0.0920
NO	1.017 (1.003, 1.031) 0.0172	1.015 (1.003, 1.028) 0.0183	1.011 (0.996, 1.026) 0.1634
P for interaction	0.0111	0.0048	0.0339
CANCER			
YES	1.007 (0.943, 1.075) 0.8366	1.1 (0.937, 1.072) 1.2 0.9503	0.996 (0.921, 1.077) 0.9187
NO	1.015 (1.002, 1.028) 0.0227	1.014 (1.001, 1.026) 0.0351	1.000 (1.000, 1.000) 0.2222
P for interaction	0.8075	0.7380	0.7286

Model 1: no covariates were adjusted. Model 2: age, gender, and race were adjusted. Model 3: age, sex, race, education level, smoking status, drinking status, BMI, diabetes, congestive heart failure,coronary artery disease, stroke and cancer were adjusted.

In the subgroup analysis stratified by gender and age, the model is not adjusted for sex and age, respectively.

Multivariate logistic regression analysis was used to calculate beta values and 95% confidence intervals.

95% CI, 95% confidence interval; OR, odds ratio; SII, systemic immunity-inflammation index;BMI, body mass index.

p< 0.05 was considered statistically significant.

**Figure 2:**
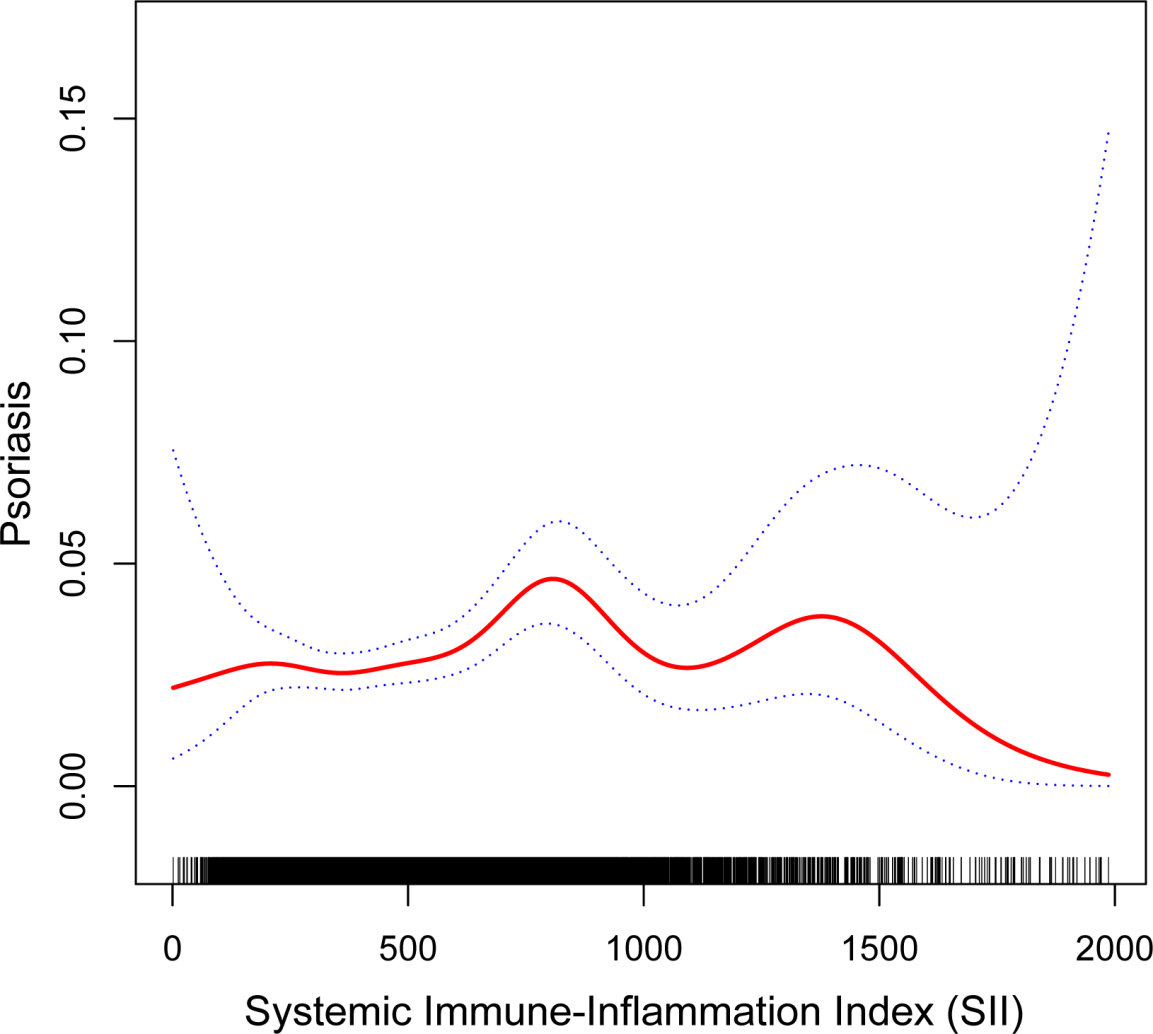
The nonlinear associations between SII and psoriasis. The solid red line represents the smooth curve fit between variables. Blue bands represent the 95% of confidence interval from the fit.

Because people with SII greater than 2000 are more discrete, data with SII greater than 2000 are removed from the curve fitting. We fitted smoothed curves to characterize the nonlinear relationship between SII and psoriasis (**Figure 2**). We found two consecutive inverted U-shaped relationships between SII and psoriasis, with the most significant inflection point of 797.067.

## 4 Discussion

The study sample utilized in our research is representative of the American population at a national level. We observed that the higher the SII participants with the greater the chance of psoriasis. Prominently, two consecutive inverted U-shaped relationships between SII and psoriasis was revealed for the first time.The risk was highest when SII was 797.067. Interestingly, the significant positive correlation between SII and psoriasis was only observed among non-Hispanic whites. Additionally, gender or weight status may have played a moderating role in the association between psoriasis and SII. There was an association in women, but not in men. The association was observed only in the obese group. In fact, Model 3 was built based on a comprehensive adjustment for all possible relevant variables, which included various factors that may influence inflammatory status, such as age, BMI, co-morbidities, and so on. If adjustments for these variables are able to attenuate or eliminate differences in SII among psoriasis patients, then the results may become less significant.

As far as we know, it is the first NHANES-based investigation of the relationship between SII and psoriasis. The link here reported regarding SII and psoriasis is similar to those previously reported, with one study from Turkey (30) showing that SII of psoriasis patients was considerably greater than that of control group and patients with moderate/severe psoriasis had higher SII than with mild psoriasis. More recently, Sugimoto et al. also demonstrated a positive correlation between SII and PASI scores and found that patients with higher SII scores had lower persistence of treatment with conventional systemic medications (31). Dincer Rota et al. reported that significantly elevated SII values were observed in both the subgroup with PASI ≥ 4.5 and among patients experiencing nail and genital involvement (32). Moreover, it has been discovered that the mean SII was lower in the patients receiving biologic treatment compared to the untreated patients (33, 34). According to an observational retrospective study, SII was positively correlated with disease severity and was an independent prognostic factor for mild and moderate psoriasis (35).

Our findings are closely related to the evidence that psoriasis is a systemic inflammatory disease (36–40). Recent research has revealed that the pathogenesis of psoriasis and its complications are related to systemic inflammatory pathways.Observations from the 18F-fluorodeoxyglucose positron emission tomography/computed Tomography study also confirmed the hypothesis that psoriasis is a systemic inflammatory disease (41). In multiple studies, in addition to marked increases in systemic arterial and subcutaneous inflammation, subclinical inflammation was also observed in the liver, joints, and tendons for individuals with moderate-to-severe psoriasis, and in the aorta of patients with mild psoriasis (42). Furthermore, insulin resistance may be more helpful in predicting subclinical atherosclerosis than traditional cardiovascular disease risk factors, and femoral artery ultrasonography better detects subclinical atherosclerosis in patients with moderate-to-severe psoriasis (43, 44). Serum levels of several proinflammatory cytokines, including TNF-α,IFN-γ,IL-6,IL-8,IL-12,IL-17A and IL-18 were elevated in individuals diagnosed with psoriasis compared to healthy controls, suggesting that psoriasis develops systemically (45).

Furthermore, the positive correlation between SII and psoriasis exhibits significant variations in relation to stroke occurrence. Extensive research has demonstrated that individuals with psoriasis face an elevated risk for cerebrovascular disease(stroke)which is attributed to the severity of their psoriasis condition (46–49). In addition, the incidence of ischemic heart disease and cerebrovascular disease increased in patients with psoriasis combined with depression (50). Numerous studies have sought to establish a causal link between psoriasis and cardiovascular disease. Cardiovascular risk factors such as dyslipidemia, diabetes mellitus, hypertension, metabolic syndrome and obesity have also increased in psoriasis patients (51). Atherosclerosis is the main pathologic change that precedes myocardial infarction and stroke. Notably, individuals with psoriasis have been found to have higher arterial stiffness than healthy controls, with arterial stiffness positively correlating with psoriasis duration (52, 53). Multiple studies measured by imaging techniques have reported a higher prevalence and greater severity of coronary artery calcification and atherosclerosis among individuals diagnosed with psoriasis in comparison to their healthy counterparts (54–56). The association between psoriasis and cardiometabolic disorders can be attributed to various mechanisms, involving the secretion of adipokines, oxidative stress, insulin resistance, angiogenesis, microparticles, hypercoagulability and common inflammatory pathways (51). Thus, the administration of effective systemic anti-inflammatory drugs may prove beneficial in reducing cardiovascular risk in psoriasis patients (57).

Platelet, neutrophil, and lymphocyte counts constitute SII. The pathogenic role of neutrophils, the most abundant innate immune cells, is associated with chronic inflammatory and autoimmune diseases (58). The abundant presence of neutrophils in psoriatic lesions is a distinctive histopathological characteristic of psoriasis (59). Recent reports have proposed that neutrophils play a role in psoriasis pathophysiology through processes such as respiratory burst, degranulation, and the formation of neutrophil extracellular traps, influencing psoriatic immunity and clinical outcomes (60). In addition, patients with psoriasis continue to consume platelets due to an enhanced coagulation response (61). Platelets play an important role in cardiovascular disease as mediators of hemostasis and thrombotic inflammation (62). Numerous studies have reported elevated levels of platelet activation biomarkers in individuals with psoriasis when compared to healthy controls. These biomarkers include platelet-derived microparticles (PDMPs) and mean platelet volume (MPV). Interestingly, a positive correlation has been observed between the levels of these biomarkers and PASI scores (63). The core pathogenesis of psoriasis involves aberrant function of multiple T-cell subsets, including regulatory T cells (Tregs), T helper (Th)1 cells, Th2 cells, Th17 cells, and Th22 cells, accompanied by aberrant release of associated cytokines, such as IFN-γ, tumor necrosis factor (TNF)-α, and members of the IL-23 and IL-17 families (45).

In recent years, with the advent of biologics, the effectiveness and safety of psoriasis treatments have greatly improved. A major challenge and limitation is to find better predictors of treatment response to determine which patients are more likely to benefit from specific biologics. SII is positively correlated with PASI scores (30–32), and the correlation with treatment response (31, 33, 34) has been confirmed by some studies. In addition, correlations have been found between SII and metabolic syndrome (64), cardiovascular disease (65), and depression (66) in psoriasis comorbidities. Taken together, SII appears to be a possible biomarker associated with psoriasis disease activity, treatment response, and the development of comorbidities, thus helping patients to develop more personalized and effective treatment strategies.

Our study has several limitations worth noting. Firstly, due to the cross-sectional design of our research, we cannot establish causality. Second, relying on self-reported questionnaire data without clinical assessment by healthcare professionals to determine the presence or absence of psoriasis may lead to misdiagnosis or underestimation of psoriasis cases, especially mild or atypical psoriasis cases. Recall bias may also affect the validity of study results. Moreover, the NHANES database does not differentiate between the various types of psoriasis and fails to obtain information on which period of disease progression (progressive/quiescent/degenerative) a psoriasis patient is in, whereas different types of psoriasis at different stages may interact biologically with SII in different ways. As it is well known that the inflammatory response is a dynamic process, high (or low) values of SII observed at a single time point may not fully reflect the complexity and dynamics of systemic inflammation. However, the interaction between inflammation and psoriasis is complex. Although we controlled for some confounders, other confounders, such as duration of psoriasis, types of psoriasis complications not included, use of biologics, and history of long-term use of anti-inflammatory medications to treat complications, may still have had an impact on the study results. Because NHANES did not document these factors, we were unable to include them in our analysis. Therefore, our results may not fully reflect the complete picture.

## 5 Conclusion

Our results establish a connection between elevated SII levels and psoriasis. SII may be a simple, practical, and easily accessible tool for monitoring disease activity and treatment efficacy in patients with psoriasis. To validate our findings, additional extensive prospective studies are warranted.

## Data availability statement

The datasets presented in this study can be found in online repositories. The names of the repository/repositories and accession number(s) can be found below: www.cdc.gov/nchs/nhanes/.

## Ethics statement

Ethical approval was not required for the study involving humans in accordance with the local legislation and institutional requirements. Written informed consent to participate in this study was not required from the participants or the participants’ legal guardians/next of kin in accordance with the national legislation and the institutional requirements.

## Author contributions

XZ: Data curation, Writing – original draft, Writing – review & editing. JL: Writing – original draft. XL: Writing – review & editing.
